# Intraoperative ketorolac in high-risk breast cancer patients. A prospective, randomized, placebo-controlled clinical trial

**DOI:** 10.1371/journal.pone.0225748

**Published:** 2019-12-04

**Authors:** Patrice Forget, Gauthier Bouche, Francois P. Duhoux, Pierre G. Coulie, Jan Decloedt, Alain Dekleermaker, Jean-Edouard Guillaume, Marc Ledent, Jean-Pascal Machiels, Véronique Mustin, Walter Swinnen, Aline van Maanen, Lionel Vander Essen, Jean-Christophe Verougstraete, Marc De Kock, Martine Berliere

**Affiliations:** 1 Institute of Applied Health Sciences, Epidemiology group, School of Medicine, Medical Science and Nutrition, University of Aberdeen, Department of Anaesthesia, NHS Grampian, Aberdeen, United Kingdom; 2 The Anticancer Fund, Brussels, Belgium; 3 Institut Roi Albert II, Service d’Oncologie Médicale, Cliniques Universitaires Saint-Luc and Institut de Recherche Clinique et Expérimentale (POLE MIRO), UCLouvain, Brussels, Belgium; 4 de Duve Institute, UCLouvain, Brussels, Belgium; 5 Department of Oncology, St-Blasius Hospital, Dendermonde, Belgium; 6 Clinical Pharmacology Unit, Cliniques Universitaires Saint-Luc, Brussels, Belgium; 7 Department of Anesthesiology, Ste-Elisabeth hospital, Namur, Belgium; 8 Department of Anesthesiology, St-Pierre Clinic, Ottignies, Belgium; 9 Department of Anesthesiology, St-Blasius Hospital, Dendermonde, Belgium; 10 Biostatistics Unit, King Albert II Institute, Cliniques Universitaires Saint-Luc, Brussels, Belgium; 11 Department of Gynecology, St-Pierre Clinic, Ottignies, Belgium; 12 Department of Anesthesiology, Centre Hospitalier Wallonie Picarde (CHWAPI), Tournai, Belgium; 13 Department of Gynecology, Breast Clinic, King Albert II Institute, Cliniques Universitaires Saint-Luc, UCLouvain, Brussels, Belgium; Tata Memorial Centre, INDIA

## Abstract

**Background:**

Ketorolac has been associated with a lower risk of recurrence in retrospective studies, especially in patients with positive inflammatory markers. It is still unknown whether a single dose of pre-incisional ketorolac can prolong recurrence-free survival.

**Methods:**

The KBC trial is a multicenter, placebo-controlled, randomized phase III trial in high-risk breast cancer patients powered for 33% reduction in recurrence rate (from 60 to 40%). Patients received one dose of ketorolac tromethamine or a placebo before surgery. Eligible patients were breast cancer patients, planned for curative surgery, and with a Neutrophil-to-Lymphocyte Ratio≥4, node-positive disease or a triple-negative phenotype. The primary endpoint was Disease-Free Survival (DFS) at two years. Secondary endpoints included safety, pain assessment and overall survival.

**Findings:**

Between February 2013 and July 2015, 203 patients were assigned to ketorolac (n = 96) or placebo (n = 107). Baseline characteristics were similar between arms. Patients had a mean age of 55.7 (SD14) years.

At two years, 83.1% of the patients were alive and disease free in the ketorolac vs. 89.7% in the placebo arm (HR: 1.23; 95%CI: 0.65–2.31) and, respectively, 96.8% vs. 98.1% were alive (HR: 1.09; 95%CI: 0.34–3.51).

**Conclusions:**

A single administration of 30 mg of ketorolac tromethamine before surgery does not increase disease-free survival in high risk breast cancer patients. Overall survival difference between ketorolac tromethamine group and placebo group was not statistically significant. The study was however underpowered because of lower recurrence rates than initially anticipated. No safety concerns were observed.

**Trial registration:**

ClinicalTrials.gov NCT01806259.

## Introduction

Non-steroidal anti-inflammatory drugs (NSAIDs) are recommended to improve pain control in the perioperative period [1]. Beyond their analgesic role, some drugs of the NSAID family, such as aspirin, may improve postoperative oncological outcomes [2, 3].

The biological effects of NSAIDs could be particularly relevant to the perioperative period, as this period is marked by an activation of inflammatory pathways, which could contribute to accelerated tumor growth and dissemination [4, 5]. In both animal models [6, 7] and retrospective studies [3, 8], perioperative administration of NSAID has been associated with lower risk of cancer recurrence. Within the NSAID family, ketorolac—routinely used during surgery—has been identified as one of the most interesting candidates to prevent recurrence in breast, lung and ovarian cancer [3, 9–11].

In the breast cancer studies, this association was particularly noted in patients at high risk of early recurrence, i.e. related to tumor-related factors (e.g. a triple-negative phenotype), signs of early dissemination (lymph-node invasion) and/or preoperative systemic inflammation as measured by the neutrophil-to-lymphocyte ratio (NLR) [3, 10, 12]. The NLR score has been proposed as a preoperative prognostic factor in multiple cancer types [13] including breast cancer [14]. In our retrospective study, a high NLR was associated with a higher risk of recurrence irrespective of the stage or of the type of breast cancer surgery [15].

Ideally, the administration of ketorolac should be limited to the shortest possible period [16], as the use of the intravenous route is limited to a few hours in case of one day surgery. A single dose of ketorolac may also be acceptable for patients with relative contraindications, such as impaired renal function, respiratory contraindication or previous digestive bleeding.

Consequently, a randomized, placebo-controlled, trial was designed to test the hypothesis that a single intraoperative dose of ketorolac may be associated with a prolonged disease-free survival after surgery in high risk breast cancer patients (NCT01806259) [15]. The primary objective was to investigate the effect of perioperative ketorolac on disease-free survival (DFS) at 2 years after breast cancer surgery.

## Patients and methods

### Study design

The study was approved by the institutional review boards of all participating centers (central ethics committee: Université catholique de Louvain, Chairperson: Jean-Marie Maloteaux, EUDRACT 2012-003774-76) and the study was conducted in accordance with the Declaration of Helsinki and applicable national and European laws. Patients provided written informed consent.

The KBCt trial was registered before patients enrollment (Principal investigator: Patrice Forget, NCT01806259, date of registration: March 7, 2013). This is a Belgian, multicenter, prospective, double-blind, placebo-controlled, randomized phase III trial in high risk breast cancer patients. Each patient was assigned to the ketorolac or the placebo group. Patients were given one dose of ketorolac tromethamine (Taradyl®, N.V. Roche S.A., Belgium) or a matching placebo. Each patient was randomly assigned on a 1:1 ratio to receive either 30 mg of ketorolac or a placebo during the induction of anesthesia (pre-incision). The placebo consisted of NaCl 0.9% (3 mL) and was identically presented to ensure double blinding. No dose modification was allowed because only one single dose was administered.

The standardized anesthetic protocol included: bolus followed by a continuous infusion of propofol (as needed to maintain bispectral index value between 40 to 60 during the surgery), ketamine 0.3 mg/kg, clonidine as needed to maintain hemodynamic stability (up to 4 μg/kg) and sufentanil by boluses of 0.1 μg/kg as needed. Airways were instrumented by a laryngeal mask airway. Postoperative analgesia included, for all the patients, the use of acetaminophen as needed (3 to 4 g/day), tramadol 50 mg (and intramuscular piritramide 10 mg, a lipophilic morphinomimetic with 0.7 of the potency of morphine, every 6 hours in the case of severe pain).

Neoadjuvant treatment, surgery and adjuvant treatment were done according to standard practices at the participating centers. Follow-up was performed by the local oncologist, every 3 months after surgery for 2 years, then every 6 months for 3 years, and at least yearly thereafter.

### Patients

Eligible patients were ≥18 and ≤75 years old with histologically or cytologically confirmed, invasive ductal or lobular breast carcinoma planned for curative breast cancer surgery. In addition, to be considered high-risk, patients had to have one of the of 3 following criteria: (i) a NLR≥4 or (ii) node-positive disease (cN1-N3) or (iii) a triple-negative phenotype. The main exclusion criteria were body weight below 50kg or above 100 kg, presence of any contra-indication to ketorolac, and a history of invasive cancer within the previous 5 years.

### Randomization, endpoints and statistical analysis

Randomization of eligible patients was done the day before surgery and used randomization blocks of 4. There was no stratification factor. In each center, a randomization list was kept accessible exclusively to the pharmacist in charge of the preparation of the study product (ketorolac or placebo). For each patient, a sealed opaque envelope was provided to permit immediate unblinding in case of emergency.

The primary endpoint of the study was Disease-Free Survival (DFS) defined as time from randomization to recurrence of invasive breast cancer; contralateral invasive breast cancer; second non-breast malignancy; or death from any cause, whichever came first [17]. Secondary endpoints were: overall survival (OS), loco-regional recurrence-free survival (LR-RFS), distant metastasis recurrence-free survival (DM-RFS), safety (intra- & post-operative blood loss, adverse events) and post-operative pain.

Based on the retrospective study and other trials in the perioperative setting [18], the study was designed to detect a 33% reduction in the risk of recurrence. With a power of 0.8 and an alpha of 0.05, 100 high risk patients per group was then needed to detect a DFS increase from 40% to 60% at 2 years.

The intention-to-treat population was used for all efficacy analyses and per-protocol population was used for sensitivity testing. Patients' demographics, baseline characteristics (including tumor characteristics and Nottingham Prognostic Index) and oncological treatments were summarized using descriptive statistics (mean, standard deviation, median, minimum and maximum values) for continuous parameters and frequencies and percentages for categorical data. DFS and OS were estimated using the Kaplan-Meier method and compared between groups by the Log-Rank test. Hazard Ratio (HR) comparing ketorolac with placebo and 95% Confidence Intervals (CIs) were estimated from a Cox proportional hazards model. Safety analyses included incidence of SAEs, intra- and postoperative bleeding. Treatment group comparisons were performed using Mann-Whitney test, Fisher or Chi-square as appropriate. All statistical tests were 2-sided with a 5% Type I error.

All data were collected using REDCap (Research Electronic Data Capture System, Vanderbilt University) and analyzed using SAS statistical software version 9.4 (Copyright, SAS Institute Inc.).

## Results

**Fig 1:**
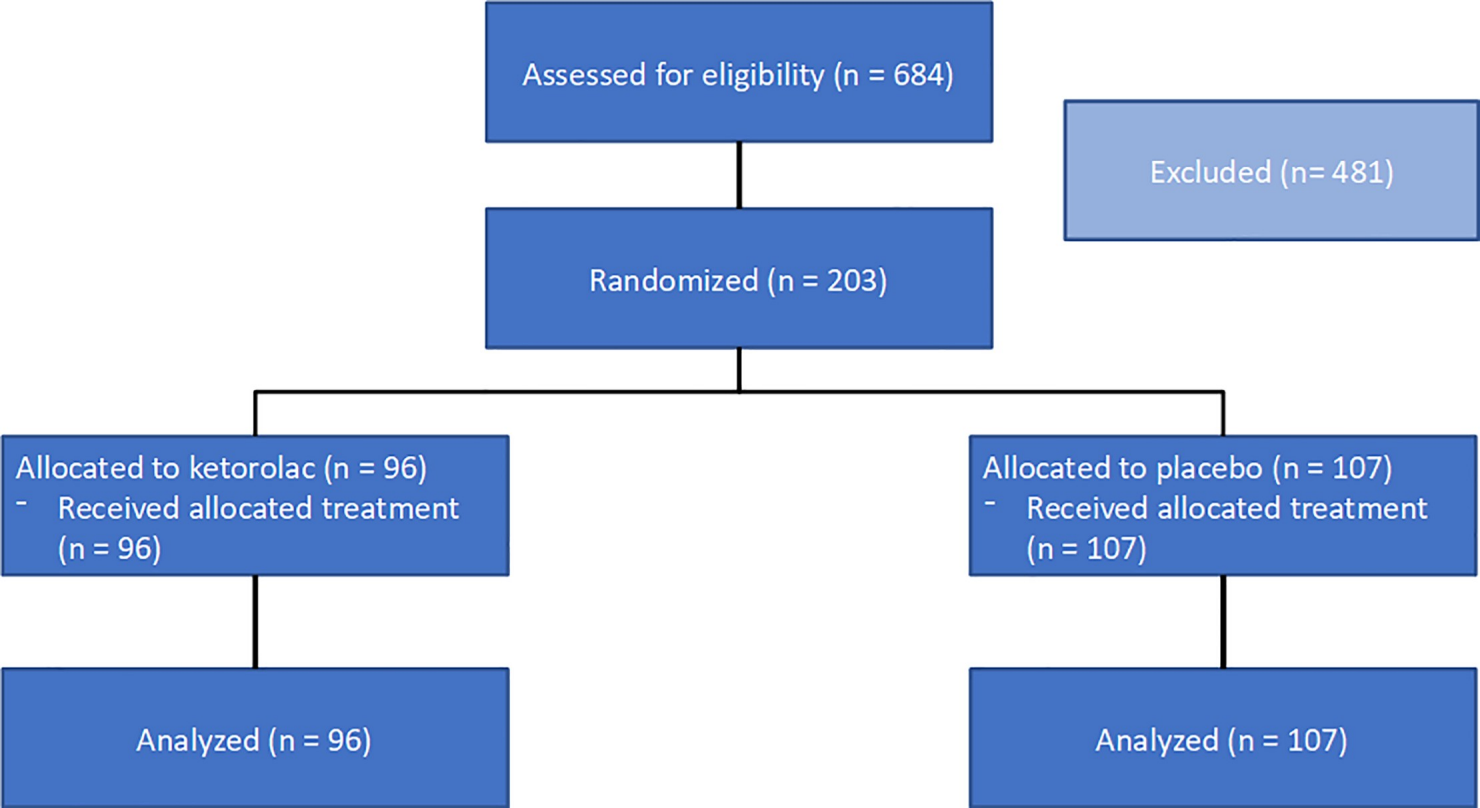
CONSORT diagram.

Between February 2013 and July 2015, 203 patients from 4 sites in Belgium were randomly assigned to ketorolac (n = 96) or placebo (n = 107) (Fig 1). A difference in patient numbers appeared due to the use of a stratified randomization (by block, by centres).

The data cutoff for the primary efficacy analysis (DFS) was February 2018 with a median follow-up of 26.9 months.

### Patients’ characteristics

Patients’ characteristics are summarized in Table 1. Baseline characteristics were similar between arms. Patients were considered at high risk of recurrence either because of a positive clinical lymph node status (n = 152), because of a triple-negative phenotype (n = 40), because of a NLR ≥4 (n = 28), or because of at least 2 of these criteria (TN & N+ (n = 10), TN & NLR (n = 3), N+ & NLR (n = 4), all 3 criteria (n = 0)). Patients had a mean age of 55.7 (SD 14) years ranging from 28 to 85. All were female except for 1 male in the ketorolac group. One patient was included despite a metastasis at diagnosis (site) and was randomized to ketorolac. This patient was included in all further analyses (intention-to-treat). All patients received the allocated treatment and all underwent surgery after randomization.

**Table 1:** Baseline characteristics of the patients, tumors, and treatments.

Characteristics	Ketorolac N = 96		Placebo N = 107	
Age, years				
Mean (SD)	56.1 (14.0)		55.4 (13.9)	
Range	30–85		28–85	
Gender, N (%)				
Female	95	(99%)	100	(100%)
Male	1	(1%)	0	(0%)
cT, N (%)				
T1	29	(30%)	28	(26%)
T2	56	(58%)	63	(59%)
T3	11	(12%)	14	(13%)
Missing	0	(0%)	2	(2%)
cN, N (%)				
N0	18	(19%)	23	(21%)
N+	78	(81%)	83	(78%)
Missing	0	(0%)	1	(1%)
cM, N (%)				
M0	92	(96%)	102	(95%)
M1	1	(1%)	3	(3%)
Missing	3	(3%)	2	(2%)
Histologic type(s), N (%)				
Invasive ductal adenocarcinoma	80	(83%)	86	(80%)
Invasive lobular adenocarcinoma	15	(16%)	12	(11%)
Other	3	(3%)	8	(8%)
Histologic grade, N (%)				
1	5	(5%)	11	(10%)
2	58	(60%)	52	(49%)
3	32	(33%)	42	(39%)
Missing	1	(1%)	2	(2%)
ER, N (%)				
Positive	66	(69%)	77	(72%)
Negative	30	(31%)	29	(27%)
Missing	0	(0%)	1	(1%)
PR, N (%)				
Positive	59	(62%)	65	(61%)
Negative	36	(37%)	42	(39%)
Missing	1	(1%)	0	(0%)
HER2/neu (IHC), N (%)				
HER2-	79	(82%)	83	(77%)
HER2+	17	(18%)	22	(21%)
Missing	0	(0%)	2	(2%)
Triple Negative, N (%)				
Yes	22	(23%)	18	(17%)
No	74	(77%)	89	(83%)
NLR >4, N (%)				
Yes	13	(14%)	15	(14%)
No	83	(86%)	92	(86%)
Chemotherapy, N (%)				
Yes	80	(83%)	81	(76%)
No	16	(17%)	26	(24%)
If chemotherapy, type (%)				
Adjuvant	27	(28%)	27	(25%)
Neoadjuvant	53	(55%)	54	(51%)
If chemotherapy, compound (%)				
Antracyclins and taxanes	80	(83%)	79	(74%)
Missing	0	(0%)	2	(2%)
If Neoadjuvant chemotherapy, compound (%)				
Antracyclins and taxanes	53	(55%)	53	(50%)
Missing	0	(0%)	1	(1%)
If Adjuvant chemotherapy, compound (%)				
Antracyclins and taxanes	27	(28%)	26	(24%)
Missing	0	(0%)	1	(1%)
Type of surgery, N (%)				
Mastectomy	60	(63%)	59	(55%)
Breast-conserving surgery	34	(35%)	48	(45%)
Missing	2	(2%)	0	(0%)
Type of lymphadenectomy, N (%)				
None	6	(6%)	8	(8%)
Sentinel	5	(5%)	7	(6%)
Complete axillary	85	(89%)	91	(85%)
Missing	0	(0%)	1	(1%)
Post-operative radiotherapy, N (%)				
Yes	77	(80%)	88	(82%)
No	19	(20%)	19	(18%)
Endocrine therapy, N (%)				
Yes	69	(72%)	69	(64%)
No	27	(28%)	37	(35%)
Missing	0	(0%)	1	(1%)
If Endocrine therapy, compound (%)				
Tamoxifen	40	(42%)	38	(36%)
Aromatase inhibitor	23	(24%)	27	(25%)
Tamoxifen before an aromatase inhibitor	6	(6%)	4	(4%)

### Efficacy

**Fig 2:**
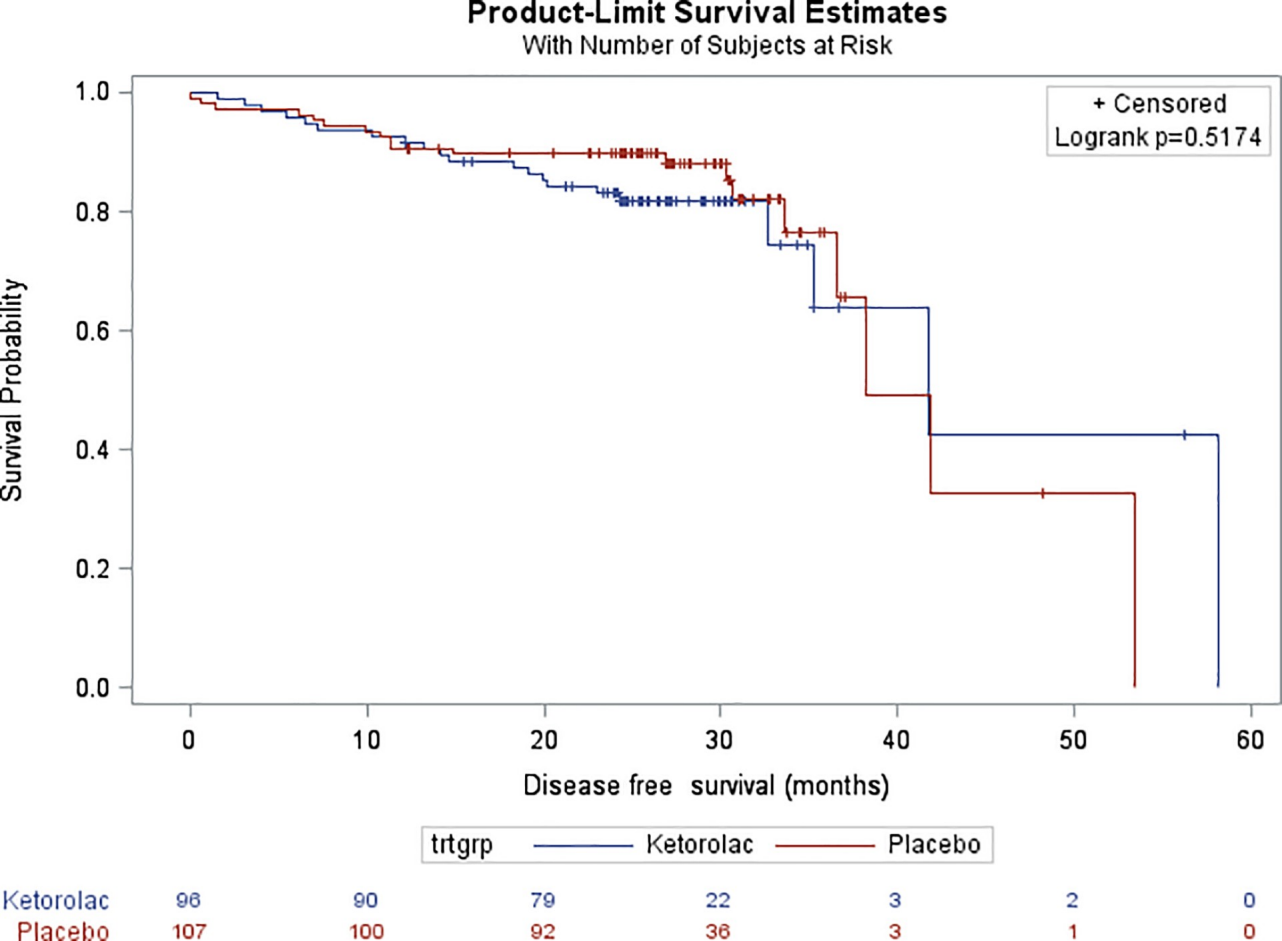
Kaplan-Meier estimates of Disease-Free Survival (DFS) in the overall study population.

After a median follow-up of 26.9 months, there was no difference in DFS (Fig 2) between groups (HR, 1.23 with reference to Placebo; 95% CI, 0.65 to 2.31; p = 0.517). At two years, 83.1% of the patients were alive and disease free in the ketorolac group compared with 89.7% in the placebo arm.

**Fig 3:**
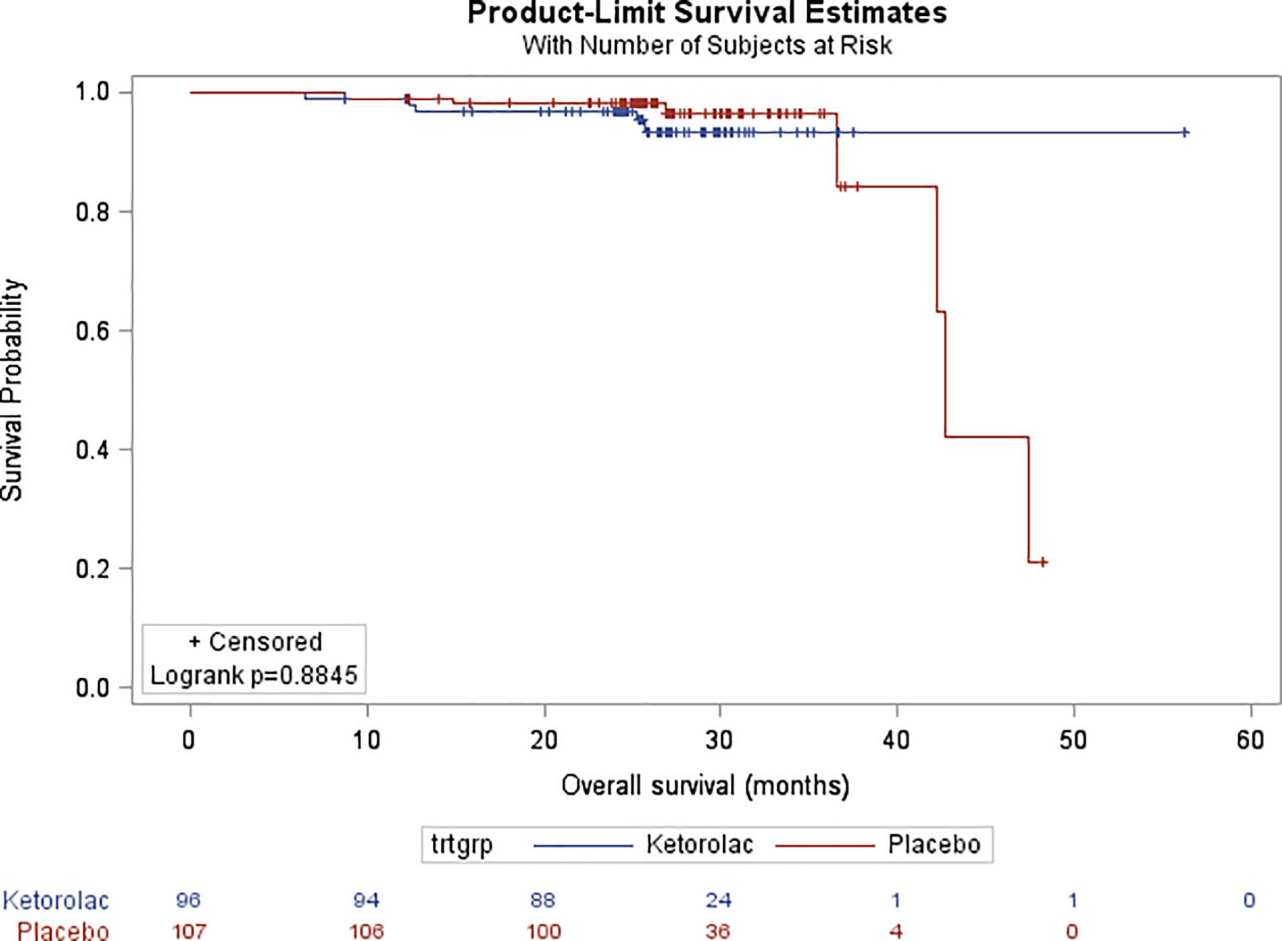
Kaplan-Meier estimates of Overall Survival (OS) in the overall study population.

There was also no difference in OS (HR, 1.09; 95% CI, 0.34 to 3.51; p = 0.884), with 96.8% of the patients who were alive after two years in the ketorolac vs. 98.1% in the placebo arm (Fig 3). Similarly, no difference was observed for LR-RFS (HR, 1.10; 95% CI,0.51 to 2.37; p = 0.816) and DM-RFS (HR, 0.26; 95% CI, 0.74 to 3.01; p = 0.255).

### Safety

A summary of perioperative events relevant to ketorolac safety profile and of all serious adverse events is presented in Table 2. There was one postoperative major bleeding in the ketorolac arm that required surgical re-intervention, which resolved the event without the need for blood transfusion. Both intra- and post-operative blood losses were not different between groups (p = 0.063 and 0.114 respectively). There was no difference in pain at D1 after surgery neither at rest (p = 0.620) nor in movement (p = 0.254).

**Table 2:** Perioperative and adverse events.

Characteristics	Ketorolac N = 96		Placebo N = 107		p-value
**Perioperative events**					
Hospital stay, days					
Mean (SD)	3.9 (1.2)		3.7 (1.0)		0.290
Range	1–8		2–7		
Intra-operative blood loss, ml					
Mean (SD)	188 (123)		176 (168)		0.063
Range	0–504		0–935		
Postoperative blood loss in drains, ml					
Mean (SD)	229 (217)		182 (164)		0.114
Range	0–1100		0–735		
Postoperative major bleeding, N (%)	1 (1%)*		0 (0%)		0.473
Pain at rest at D1 after surgery Verbal simple scale (0–4), N (%)					0.620
0	18	(19%)	17	(16%)	
1	35	(37%)	31	(29%)	
2	37	(38%)	47	(44%)	
3	5	(5%)	10	(9%)	
4	1	(1%)	1	(1%)	
Missing	0	(0%)	1	(1%)	
**Adverse events**					
Any serious adverse event	8	(8%)	7	(7%)	0.789

* 1 patient in the ketorolac arm experienced major bleeding at the surgical site that necessitated re-intervention. No transfusion was necessary and the problem resolved after re-intervention

## Discussion

This study shows that a single administration of 30 mg of ketorolac tromethamine does not improve disease-free survival in high-risk breast cancer patients. Overall survival is also comparable between the two groups. No safety concerns were raised in this study.

We selected patients known to carry a higher risk of recurrence, namely patients with a triple negative phenotype, patients with node involvement or patients with a high NLR. The assumptions about the recurrence rate made when calculating the sample size were unfortunately not respected, which led to our study being underpowered. However, the survival curves are superimposable, which supports the conclusion of a lack of effect of a single administration of ketorolac to prevent breast cancer recurrence.

What remains is that several studies indicates that inflammatory pathways are implicated in postoperative cancer recurrences [5, 6, 19]. The surgical stress activates numerous pathways known to promote tumor growth and one possible way to intervene is to use NSAIDs, like ketorolac [20]. But, since nearly all anesthetic and analgesic drugs affect anticancer immunity and other tumor-promoting pathways (such as angiogenesis and VEGF) [21–23], ketorolac represents only one of the perioperative drug candidates to reduce the risk of recurrence [5, 24]. For instance, it could be that targeting both catecholamines and prostaglandins is necessary to prevent the pro-metastatic processes induced by surgical stress as shown in preclinical experiments in several models [25]. A clinical trial using propranolol—a non-selective beta-blocker—and etodolac—an NSAID—in breast cancer patients provided encouraging biological results but could not assess the impact of this approach on recurrence because of a limited size [26].

Bimodality in the relapse frequency over time in early stage breast cancer also remains a reality [27]. An early peak, occurring in the first 18 postoperative months, has been observed repeatedly in breast cancer patients [28–30] with particular relevance in patients with large tumor size, high histological grade, lymph node involvement or low expression of estrogen receptors [12, 31]. However, the occurrence of the early peak is not here confirmed. Moreover, a recent retrospective study found that the anticancer effect of ketorolac may be particularly prominent in patients with a high Body Mass Index (BMI) [32]. However, we could not confirm this hypothesis in this prospective trial (data not shown), as no difference in outcomes was observed when stratifying the analyses per BMI group (≤25 vs. >25)—keeping in mind that patients weighting more than 100 kg were excluded from the trial. Taken together, it appears that new works are needed to investigate the patients’ subgroups that may specifically benefit from intraoperative interventions during cancer surgery.

If a single dose of ketorolac has no impact on recurrence, it does not preclude an effect of a longer administration. However, our choice of testing a single administration was partly supported by the increased risk of adverse events with prolonged administration of ketorolac [16]. Further studies with ketorolac may focus on the identification of relevant biomarkers, and its effect on these biomarkers in specific patient subgroups. Because multiple biological pathways are modulated by ketorolac (through the inhibition of both the COX-2 and the COX-1 enzymes [33] but also independently [11]), selecting a population more likely to benefit of a longer administration of ketorolac might however be difficult.

In summary, these data do not support the use of a single administration of ketorolac before breast cancer surgery to prevent breast cancer recurrence. To progress further, pivotal trials may be performed, focusing on the identification of relevant biomarkers, and the effect of ketorolac on these biomarkers in specific patient subgroups.

## Supporting information

S1 File Consort checklist. (DOC)

S2 File Data. (XLSX)

S3 File Protocol. (DOC)
