# Supplementary material for: Intraoperative ketorolac in high-risk breast cancer patients. A prospective, randomized, placebo-controlled clinical trial
Source: PLoS One. 2019 Dec 4;14(12):e0225748. doi: 10.1371/journal.pone.0225748 (PMC6892544; doi:10.1371/journal.pone.0225748)
Supplement: S3 File — (DOC) [file pone.0225748.s003.doc]

###### Statistical Analysis Plan

###### Protocol 2012-003-774-76/ KBCt

**Perioperative ketorolac in cancer patients with inflammation.**

**Rationale, feasibility and methodology of a prospective randomized trial.**

Version: Draft

Date: 23^rd^ October 2017

Statistical Analysis Plan Signature Page

**Promotor: Cliniques universitaires Saint-Luc**

###### Protocol: Protocol 2012-003-774-76/ KBCt

###### Title: Perioperative ketorolac in cancer patients with inflammation.

###### Rationale, feasibility and methodology of a prospective randomized trial.

**Status: Draft**

**Date: 23^rd^ October 2017**

Prepared by:

_____________________________________________________________________________

**Aline van Maanen, Biostatistician Date­­­­**

Statistical Support Unit,

King Albert II Institute,

Cliniques universitaires Saint-Luc

<Merci de confirmer tous les signataires et leur affiliation>

Approved by:

_____________________________________________________________________________

**Patrice Forget, Study Coordinator Date**

Vrije Universiteit Brussel (VUB), Universitair Ziekenhuis Brussel (UZ Brussel), Anesthesiology and Perioperative Medicine

_____________________________________________________________________________

Prof Marc DE KOCK**, Date**

Centre Hospitalier de Wallonie Picarde (CHWAPI), Department of Anesthesiology

_____________________________________________________________________________

**Prof. Martine Berlière, Date**

King Albert II Institute and Breast Clinic,

Cliniques universitaires Saint-Luc

and Institut de Recherche Expérimentale et Clinique, Université catholique de Louvain

_____________________________________________________________________________

**Prof. François Duhoux Date**

King Albert II Institute and Department of Medical Oncology,

Cliniques universitaires Saint-Luc

and Institut de Recherche Expérimentale et Clinique, Université catholique de Louvain

____________________________________________________________________________

**Prof. Jean-Pascal Machiels Date**

King Albert II Institute and Department of Medical Oncology,

Cliniques universitaires Saint-Luc

and Institut de Recherche Expérimentale et Clinique, Université catholique de Louvain

_____________________________________________________________________________

**Prof. Pierre Coulie Date**

de Duve Institute

Université catholique de Louvain

Table of Contents

[1 INTRODUCTION 7](#__RefHeading___Toc496538152)

[2 ABBREVIATIONS 7](#__RefHeading___Toc496538153)

[3 STUDY OBJECTIVES 7](#__RefHeading___Toc496538154)

[3.1 Primary Objectives 7](#__RefHeading___Toc496538155)

[3.2 Secondary Objectives 7](#__RefHeading___Toc496538156)

[4 STUDY DETAILS 8](#__RefHeading___Toc496538157)

[4.1 Overall Study Design 8](#__RefHeading___Toc496538158)

[4.2 Study Events Flow Chart 8](#__RefHeading___Toc496538159)

[4.3 Interim Analysis 9](#__RefHeading___Toc496538160)

[4.4 Data Safety Monitoring Board 9](#__RefHeading___Toc496538161)

[4.5 Sample Size Determination 9](#__RefHeading___Toc496538162)

[4.6 Randomization 10](#__RefHeading___Toc496538163)

[5 PERSONNEL 10](#__RefHeading___Toc496538164)

[6 ANALYSIS POPULATIONS 10](#__RefHeading___Toc496538165)

[6.1 Full Analysis Set 10](#__RefHeading___Toc496538166)

[6.2 Per Protocol Set 10](#__RefHeading___Toc496538167)

[6.3 Safety Analysis Set 11](#__RefHeading___Toc496538168)

[7 GENERAL STATISTICAL METHODOLOGY 11](#__RefHeading___Toc496538169)

[7.1 General Statistical Conventions 11](#__RefHeading___Toc496538170)

[7.2 Covariates 11](#__RefHeading___Toc496538171)

[7.3 Visit Windows 12](#__RefHeading___Toc496538172)

[7.4 Missing Dates 12](#__RefHeading___Toc496538173)

[7.4.1 Recurrence Date 12](#__RefHeading___Toc496538174)

[7.4.2 Death Date 12](#__RefHeading___Toc496538175)

[8 EFFICACY VARIABLES 12](#__RefHeading___Toc496538176)

[9 SAFETY VARIABLES 15](#__RefHeading___Toc496538177)

[10 PLANNED STATISTICAL METHODS 16](#__RefHeading___Toc496538178)

[10.1 Patient Disposition 16](#__RefHeading___Toc496538179)

[10.2 Demographic and Baseline Characteristics 16](#__RefHeading___Toc496538180)

[10.3 Pre-operative, concomitant and post-operative pain medications 16](#__RefHeading___Toc496538181)

[10.4 During surgery information 16](#__RefHeading___Toc496538182)

[10.5 Chemo-, hormone-, or other anti-neoplastic therapy information 16](#__RefHeading___Toc496538183)

[10.6 Post-operative radiotherapy information 17](#__RefHeading___Toc496538184)

[10.7 Pain scales 17](#__RefHeading___Toc496538185)

[10.8 Hospital discharge 17](#__RefHeading___Toc496538186)

[10.9 Primary and Secondary Efficacy Analyses 17](#__RefHeading___Toc496538187)

[10.9.1 Primary analysis 17](#__RefHeading___Toc496538188)

[10.9.2 Secondary analyses 17](#__RefHeading___Toc496538189)

[10.10 Safety Analyses 17](#__RefHeading___Toc496538190)

[10.10.1 Serious Adverse events 17](#__RefHeading___Toc496538191)

[10.10.2 Cardiac and gastro-intestinal events 18](#__RefHeading___Toc496538192)

[10.10.3 Chronic pain 18](#__RefHeading___Toc496538193)

[10.10.4 Post-operative bleeding 18](#__RefHeading___Toc496538194)

[10.10.5 Laboratory parameters 18](#__RefHeading___Toc496538195)

[11 APPENDICES 18](#__RefHeading___Toc496538196)

[10.1 Appendix I – Table, Figure and Listing Templates 18](#__RefHeading___Toc496538197)

[10.2 Appendix II – Banned medications 18](#__RefHeading___Toc496538198)

# INTRODUCTION

###### The purpose of this document is to describe the statistical methods, data derivations and data summaries to be employed in the study entitled “Perioperative ketorolac in cancer patients with inflammation - Rationale, feasibility and methodology of a prospective randomized trial”.

The preparation of this statistical analysis plan (SAP) has been based on International Conference on Harmonisation (ICH) E3 and E9 Guidelines and in reference to Protocol 2021-003-774-76 / KBCt, November 25^th^, 2012, version 2.5.

.

# ABBREVIATIONS A COMPLETER

The following abbreviations will be used within this SAP.

DFS Disease-Free Survival

DSMB Data Safety Monitoring Board

FAS Full Analysis Set

LFS Locoregional-Free Survival

MFS Metastasis-Free Survival

OS Overall Survival

PP Per-Protocol Analysis Set

RFS Recurrence-Free Survival

SAE Serious Adverse Event

SAP Statistical Analysis Plan

SAS® SAS® Software

# STUDY OBJECTIVES

### Primary Objectives

- The primary objective of this study is to evaluate the effect of perioperative ketorolac on disease-free survival (DFS) after at least 2 years of follow-up in high risk breast cancer patients.

### 3.2 Secondary Objectives

The secondary objectives are as follows:

- To examine the effect of perioperative ketorolac on LFS at 2 and 5 years in high risk breast cancer patients.
- To examine the effect of perioperative ketorolac on Metastasis-Free Survival (MFS).
- To examine the effect of perioperative ketorolac on Overall Survival (OS)
- To examine the effect of perioperative ketorolac on post-operative pain and peri- and post-operative blood loss.
- To determine the safety profile of perioperative ketorolac based on serious adverse events (SAEs)

# STUDY DETAILS

### 4.1 Overall Study Design

This is a national, multicenter, prospective, double-blind, placebo-controlled, randomized phase III trial of peri-operative ketorolac in high risk breast cancer patients.

Patients providing written informed consent will be assessed at a screening visit to determine their eligibility for the trial. Those eligible will be asked to attend the trial center at baseline, corresponding to the day of surgery. Each patient will be assigned to one group (ketorolac or placebo), and 1 dose of ketorolac (30 mg) or placebo, identically presented, will be attributed to the patient for ulterior administration. A closed envelope joined to the medications will permit potential immediate unblinding for emergency cases. In both groups, the administration of the study drug (ketorolac or placebo) will be done during the induction of anesthesia (pre-incision).

### 4.2 Study Events Flow Chart

Table A provides a detailed description of the schedule of events.

Table A: Schedule of events

|  | **Screening** | **Treatment** | **Hospital discharge** | **Follow-up at 1, 2, 5 years** |
| --- | --- | --- | --- | --- |
|  | **D-21 to D0** | **D0** | **D8** | **1, 2, 5 years** |
| Written consent | X |  |  |  |
| Inclusion/exclusion criteria | X | X |  |  |
| Randomization |  |  |  |  |
| Baseline data | X |  |  |  |
| Medications | X |  | X | X |
| Histology |  |  | X | X |
| Hospital discharge |  |  | X |  |
| Follow-up at 1 year |  |  |  | X |
| Follow-up at 2 years |  |  |  | X |
| Follow-up at 5 years |  |  |  | X |
| Chemotherapy |  |  | X | X |
| Radiotherapy |  |  | X | X |
| Chronic pain |  |  |  | X |
| Locoregional recurrence assessment |  |  |  | X |
| Distant metastases |  |  |  | X |
| Tumor biomarkers |  |  |  | X |
| Cardiovascular assessment |  | X | X | X |
| Gastro-intestinal assessment |  | X | X |  |
| Blood loss |  | X | X |  |
| Serious Adverse Event |  | X | X |  |
| Death information | X | X | X | X |
| Unblinding |  | X |  |  |
| Screening Log | X |  |  |  |

### 4.3 Interim Analysis

No interim analysis will be performed for this study.

### 4.4 Data Safety Monitoring Board

No Data Safety Monitoring Board (DSMB) meeting will be performed for this study.

### 4.5 Sample Size Determination

Sample size estimation is presented in the protocol. Patient numbers were based on the following assumptions:

Based on a reduction of recurrence of 33%, with a type I error of 5% and a power of 80%, the number of patients eligible is of 200 (Log Rank test), randomized in a 1:1 ratio between ketorolac and placebo.

### 4.6 Randomization

This is a double blind study. Eligible patients will be randomized into the treatment arms with a ratio of 1:1 (ketorolac: placebo) as follows: Randomization will be performed 2 to 4h before surgery. A number will be assigned to each patient, as well as a treatment number, corresponding to ketorolac (30mg) or placebo (NaCl 0.9% 3 mL). Both are identical looking. In case of emergency, an unblinding envelope will be available. In both treatment groups, the randomized treatment will be administered by slow IV infusion at the induction of anesthesia.

The randomization will not be stratified.

# PERSONNEL

The statistical analysis of the study will be conducted by the statistical support unit of the King Albert II Institute.

# ANALYSIS POPULATIONS

In accordance with recommendations depicted in the ICH E9 guideline, the following analysis populations are defined as follows:

### 6.1 Full Analysis Set

The Full Analysis Set (FAS) population is defined as all subjects who have been randomized into the study, whether they actually received treatment or not. Subjects will be analyzed according to the treatment group to which they were randomized. This corresponds to the intent-to-treat (ITT) principle. The FAS population will be the set used for all the efficacy analyses, unless otherwise specified.

### 6.2 Per Protocol Set

The Per Protocol (PP) Set population will comprise all subjects who qualify for the FAS and who do not have any of the following major protocol deviations:

Violated the study entry criteria

Have taken banned concomitant medications (list available in Appendix II)

Did not receive treatment as per protocol (mis-randomized)

Potential protocol violators will be identified and reviewed to determine whether they will be excluded from the PPS population. This will take place in a review meeting prior to database lock. The above list is not an exclusive list of major violations. Other violations may be identified during the review. Formal sign-off of the final list of patients to be excluded from the Per Protocol analysis will take place after database lock. Every effort will be made to ensure that decisions will not be influenced by the treatment group to which the patient has been randomized. This analysis population will be used to support the primary efficacy analysis.

### 6.3 Safety Analysis Set

The Safety Analysis Set population will include all subjects who have taken at least one dose of study medication. Subjects will be analyzed according to the study medication they received. The safety population will be used for all safety analyses.

# GENERAL STATISTICAL METHODOLOGY

### 7.1 General Statistical Conventions

The following general statistical conventions will be implemented, unless the statistical analysis plan or protocol specify otherwise:

- For continuous variables, descriptive statistics will include the number of subjects (n), mean, standard deviation, minimum, median and maximum. For categorical data, frequency counts and percentages will be presented.
- Analysis of all efficacy and safety variables will be based on observed data
- Day converts to month: use number of days divided by 30.4
- Day converts to year: use number of days divided by 365.25
- If more than one measurement of a parameter is performed during the screening period, then the last of all such measurements prior to treatment will be considered the “baseline” value for that patient.
- All data processing, summarization and analyses will be performed using SAS®, Version 9.4 or later.

### 7.2 Covariates

No stratification factors will be considered in efficacy analyses as covariates.

### 7.3 Visit Windows

Tumor assessments will be followed at least every year, according to standard practice.

### 7.4 Missing Dates

#### 7.4.1 Recurrence Date

In the unlikely event that a patient’s date of recurrence is completely missing or partially missing, the date of progression will be treated as described in this section to estimate the time to events in the efficacy analyses:

- - If the month and year of recurrence are known (i.e. only the day is missing), the patient will be censored at the last documented scheduled tumor assessment closest to the month.
  - If only the year of recurrence is known (i.e. both the day and the month are missing), the patient will be censored at the last documented scheduled tumor assessment within the year or at the last documented scheduled visit in the previous year if there is no documented tumor assessment in the year.
  - If the date of recurrence is completely missing, the patient will be censored at the last documented scheduled tumor assessment

#### 7.4.2 Death Date

In the unlikely event that a patient’s death date is missing, the date of death will be assumed as described in this section in calculating the time to events (DFS, LFS, MFS or OS) in the efficacy analyses:

- - If the information of date of death is partially or completely missing, the patient will be censored at the last documented information obtained for living patient.

# EFFICACY VARIABLES

Events for LFS will mean CRF 20 OR 99 = Yes,

- Locoregional recurrence-free survival (LFS)

A patient’s locoregional survival time is defined as the time from surgery to the date of locoregional recurrence/death from any cause. In the case of patients who did not present a locoregional recurrence at the time of data-cut-off, the survival time will be censored.

| LFS (days) | = Date of locoregional  recurrence / Death/  Censoring | - Date of surgery + 1 |
| --- | --- | --- |
|  |  |  |

The censoring rules used in the calculation of LFS will be obtained from Table 1 below.

| **Table 1. Censoring rules used in the calculation of LFS** | | |
| --- | --- | --- |
| **Situation** | **End date** | **Censored** |
| Documented locoregional recurrence during the study | Date of the 1^st^ assessment of the series of tests that determined locoregional recurrence | No^a^ |
| Death during the study before locoregional recurrence | Date of death | No |
| Locoregional recurrence documented between scheduled visits | Date of the 1^st^ assessment of the series of tests that determined locoregional recurrence | No |
| Discontinued due to locoregional recurrence, but no documented locoregional recurrence. | Date of last tumor assessment before discontinuation. | Yes |
| No baseline assessments | Date of surgery | Yes |
| Death or locoregional recurrence after one or more missed scheduled assessments | Date of last tumor assessment | Yes |
| Patients still on treatment without PD as of data cut-off | Date of last tumor assessment | Yes |

^a^ Earliest date among the dates used in calculating the progression-free survival.

- Disease-free survival (DFS)

A patient’s DFS is defined as the time from surgery to the date of disease recurrence/death from any cause. In the case of patients who did not present any event (i.e. those lost to FU or patients who did not have any recurrence at the time of data cut-off), DFS will be censored.

| DFS (days) | = Date of recurrence /  Death/ Censoring | – Date of surgery + 1 |
| --- | --- | --- |

The censoring rules used in the calculation of DFS will be obtained from Table 2 below.

| **Table 2. Censoring rules used in the calculation of DFS** | | |
| --- | --- | --- |
| **Situation** | **End date** | **Censored** |
| Documented recurrence during the study | Date of the 1^st^ assessment of the series of tests that determined recurrence | No^a^ |
| Death during the study before recurrence | Date of death | No |
| Recurrence documented between scheduled visits | Date of the 1^st^ assessment of the series of tests that determined recurrence | No |
| Discontinued due to recurrence, but no documented recurrence. | Date of last tumor assessment before discontinuation. | Yes |
| No baseline assessments | Date of surgery | Yes |
| Death or recurrence after one or more missed scheduled assessments | Date of last tumor assessment | Yes |
| Patients without recurrence as of data cut-off | Date of last tumor assessment | Yes |

^a^ Earliest date among the dates used in calculating the progression-free survival.

How can this be found ? Using CRF 20, 30 OR 99 = Yes.

- Metastasis-free Survival (MFS)

A patient’s MFS is defined as the time from surgery to the date of metastasis occurence/death from any cause. In the case of patients who did not present an event (i.e. those lost to FU or patients who did not have any metastases at the time of data-cut-off), MFS will be censored.

| MFS (days) | = Date of metastasis /  Death/ Censoring | – Date of surgery + 1 |
| --- | --- | --- |

The censoring rules used in the calculation MFS will be obtained from Table 3 below.

| **Table 3. Censoring rules used in the calculation of MFS** | | |
| --- | --- | --- |
| **Situation** | **End date** | **Censored** |
| Documented metastases during the study | Date of the 1^st^ assessment of the series of tests that determined metastases | No^a^ |
| Death during the study before metastases | Date of death | No |
| Metastases documented between scheduled visits | Date of the 1^st^ assessment of the series of tests that determined metastases | No |
| Discontinued due to metastases, but no documented recurrence. | Date of last tumor assessment before discontinuation. | Yes |
| No baseline assessments | Date of surgery | Yes |
| Death or metastases after one or more missed scheduled assessments | Date of last tumor assessment | Yes |
| Patients without metastases as of data cut-off | Date of last tumor assessment | Yes |

^a^ Earliest date among the dates used in calculating the progression-free survival.

How can this be found ? Using CRF30, 99 = Yes.

- Overall Survival (OS) (event: CRF 99 = Yes)

A patient’s survival time will be defined as the time from surgery to the date of death due to any cause. If the patient has not died, the survival time will be censored on the last date the patient was known to be alive.

| Overall Survival (days) | = Date of death / censoring | - Date of surgery + 1 |
| --- | --- | --- |

# SAFETY VARIABLES

The safety variables include:

- - post-operative pain (CRF pain scales, post-operative pain medications and CRF16 chronic pain)
  - peri- and post-operative blood loss. (<note to programmer: CRF perioperative blood loss and CRF60)
  - Serious adverse events (note to programmer: CRF70)

Cardiac events (CRF40) and gastro-intestinal events (CRF50).

A Serious Adverse Event (SAE) is defined as any AE that results in death, is life threatening, requires inpatient hospitalization or prolongation of existing hospitalization, results in persistent or significant disability/incapacity, or is a congenital anomaly/birth defect. Drug relationship will be assessed as above.

SAEs will be considered only during the 30 days after the administration of the study product (single administration, first=last).

In the event that a partial Start Date (month/year or year) is available, and month/year or year occurs before that of first treatment dose, the AE is considered as non-treatment emergent. However, if month/year or year occurs on or after the date of first treatment dose, the following cases will be considered:

- if month/year is available and month/year of Start Date >= month/year of first treatment dose, the AE is treatment emergent and Start Date will be estimated as the first day of the month;
- if only year is available and year of Start Date >= year of first treatment dose, the AE is treatment emergent and Start Date will be estimated as Jan. 1 of that year.
- In the event that the Start Date is completely missing, Stop Date will be used to determine when the AE resolved. If the AE stopped before first treatment dose, the AE will be considered as non-treatment emergent; otherwise, if the AE stopped on or after first treatment dose or is ongoing, the AE will be considered as treatment emergent and the Start Date of the event will be estimated as first treatment dose.

The estimated start dates will not be listed.

# PLANNED STATISTICAL METHODS

Efficacy analyses will be reported overall and split by the treatment groups (ketorolac, placebo). Other specific details on the handling of the particular endpoints are provided below. Full details of the tables, figures and listing that will be produced are given in Appendix I.

<Please confirm whether listings are required in addition to tables and figures>

### 10.1 Patient Disposition

A complete accounting of patient eligibility and treatment allocation will be tabulated overall and by treatment group. The purpose of these tables is to account for patient participation in the analysis populations. Supportive listings will be provided.

### 10.2 Demographic and Baseline Characteristics

Demographic characteristics such as patient age, gender, BMI, histopathology will be tabulated overall and by treatment group for the FAS population. All continuous data will be summarized using descriptive statistics (mean, standard deviation, median, minimum and maximum values). All categorical data will be summarized using frequencies and percentages. Supportive listings will be provided.

### 10.3 Pre-operative, concomitant and post-operative pain medications

Pre- and post-operative pain medications will be tabulated overall and by treatment group for the FAS population. Supportive listings will be provided.

### 10.4 During surgery information

Hemodynamics and peri-operative blood losses will be tabulated overall and by treatment group for the FAS population. Supportive listings will be provided.

### 10.5 Chemotherapy, endocrine therapy, or other anti-neoplastic therapy information

Chemotherapy, endocrine therapy, or other anti-neoplastic therapy information will be tabulated overall and by treatment group for the FAS population. Supportive listings will be provided.

### 10.6 Post-operative radiotherapy information

Post-operative radiotherapy information will be tabulated overall and by treatment group for the FAS population. Supportive listings will be provided.

### 10.7 Pain scales

Pain scales will be tabulated overall and by treatment group for the FAS population at the various timepoints after surgery. Supportive listings will be provided.

### 10.8 Hospital discharge

Whether the patient was discharged home or not will be tabulated overall and by treatment group for the FAS population. Supportive listings will be provided.

### 10.9 Primary and Secondary Efficacy Analyses

#### 10.9.1 Primary analysis

The DFS will be summarized by treatment group. 95% confidence intervals will also be provided. The effect of ketorolac will be assessed by comparing the 2 treatment groups by a log rank test.

Potential need for adjustment regarding the inclusion criteria defining the ‘high risk’ profile of the patient, i.e high NLR AND/OR lymph nodes invasion AND/OR triple negative histopathology.

This summary will be reported using the FAS population and repeated for the PP population.

#### 10.9.2 Secondary analyses

The 5 years locoregional RR will be analyzed in a similar way as the primary analysis.

Locoregional RFS, DFS, MFS and OS will be summarized using Kaplan-Meier plots. The Kaplan-Meier estimate of the event times will be presented with 95% confidence intervals. These summaries will be done on the FAS population.

The effect of ketorolac will be assessed by comparing the 2 treatment groups by a log-rank test.

.

### 10.10 Safety Analyses

All summaries will be performed on the Safety Analysis Set population.

#### 10.10.1 Serious Adverse Events

The number of events and number and percentage of patients with SAEs will be summarized by treatment group and overall. Similar summaries will be produced for: drug-related SAEs, SAEs by maximum severity grade, SAEs leading to death. These summaries will be tabulated overall and by treatment group for the FAS. Supportive listings will be provided.

#### 10.10.2 Cardiac and gastro-intestinal events

Cardiac and gastro-intestinal events will be tabulated overall and by treatment group. Similar summaries will be produced for events leading to death. Supportive listings will be provided.

#### 10.10.3 Chronic pain

Pain scales will be tabulated overall and by treatment group. Supportive listings will be provided.

#### 10.10.4 Post-operative bleeding

Post-operative bleeding will be tabulated overall and by treatment group. Supportive listings will be provided.

#### 10.10.5 Laboratory parameters

CA 15.3 collected at baseline and treatment visits will be summarized by visit for each treatment group and overall. Change from baseline will be reported if baseline was above the upper limit of normal. If baseline was below the upper limit of normal, change to from below to above upper limit of normal will be reported.

Each data will be categorized as Normal, High, Low according to laboratory normal ranges. Shift tables will be produced with frequency counts showing the changes from the baseline visit. Supportive listings will be provided.

Biomarkers data elevation will also be reported.

# APPENDICES

### 10.1 Appendix I – Table, Figure and Listing Templates

See attached document <Will be provided when comments on content of SAP will be received>

### 10.2 Appendix II – Banned medications

See attached document <To be provided >
